# An Overview of Endometrial Cancer with Novel Therapeutic Strategies

**DOI:** 10.3390/curroncol30090574

**Published:** 2023-08-27

**Authors:** Theresa M. Kuhn, Saeeda Dhanani, Sarfraz Ahmad

**Affiliations:** 1 Gynecologic Oncology Program, AdventHealth Cancer Institute, Orlando, FL 32804, USA; 2 Philadelphia College of Osteopathic Medicine, Suwanee, GA 30024, USA

**Keywords:** endometrial cancer, hyperplasia, diagnosis, prognosis, treatment

## Abstract

Endometrial cancer (EC) stands as the most prevalent gynecologic malignancy. In the past, it was classified based on its hormone sensitivity. However, The Cancer Genome Atlas has categorized EC into four groups, which offers a more objective and reproducible classification and has been shown to have prognostic and therapeutic implications. Hormonally driven EC arises from a precursor lesion known as endometrial hyperplasia, resulting from unopposed estrogen. EC is usually diagnosed through biopsy, followed by surgical staging unless advanced disease is expected. The typical staging consists of a hysterectomy with bilateral salpingo-oophorectomy and sentinel lymph node biopsies, with a preference placed on a minimally invasive approach. The stage of the disease is the most significant prognostic marker. However, factors such as age, histology, grade, myometrial invasion, lymphovascular space invasion, tumor size, peritoneal cytology, hormone receptor status, ploidy and markers, body mass index, and the therapy received all contribute to the prognosis. Treatment is tailored based on the stage and the risk of recurrence. Radiotherapy is primarily used in the early stages, and chemotherapy can be added if high-grade histology or advanced-stage disease is present. The risk of EC recurrence increases with advances in stage. Among the recurrences, vaginal cases exhibit the most favorable response to treatment, typically for radiotherapy. Conversely, the treatment of widespread recurrence is currently palliative and is best managed with chemotherapy or hormonal agents. Most recently, immunotherapy has emerged as a promising treatment for advanced and recurrent EC.

## 1. Introduction

The endometrium is a tissue found in the inner mucosal lining of the uterus that prepares for potential implantation and provides nutritional support for pregnancy. This is carried out by cycling between shedding, regeneration, and differentiation in response to hormonal, vascular, and stromal influences [1,2]. Estrogen and progesterone work in a tightly co-ordinated interaction that is dependent upon conception and the production of human chorionic gonadotropin (hCG). With the presence of both, estrogen promotes the growth of the endometrium, whereas, in its absence, there is a production of progesterone, which leads to endometrial shedding. This allows menses to occur. The potential for neoplastic processes occurs when there is continuous, unopposed estrogen production in the proliferative, hyperplastic endometrium. However, not all endometrial carcinoma arises from a hormone-sensitive background and can also arise from genetic alterations.

Uterine cancer was expected to affect 66,200 women in the United States in 2023, which makes up 7% of all women cancers [3]. Of all the gynecologic malignancies, endometrial carcinoma represents the most common in developed countries [4]. It was once classified based on its hormone sensitivity in the type 1 (estrogen-dependent, endometrioid) and type 2 (estrogen-independent, non-endometrioid, i.e., serous or clear cell) systems proposed by Bokhman in 1983 [5]. This traditional approach came with an associated epidemiological background, which was thought to assist healthcare providers in making a timely, accurate diagnosis. Those who fit the criteria for type 1 tended to be obese, had a history of either chronic anovulation or estrogen hormone-replacement therapy, were highly sensitive to progestogens, presented at a low grade and in an early stage, and had a favorable prognosis [5]. Those who were not hormone sensitive (type 2) were older, had no history of excess estrogen, had decreased sensitivity to progestogens, presented at a high grade and a late stage, and had a poor prognosis [5,6].

In 2013, The Cancer Genome Atlas (TCGA) redefined the classification of endometrial carcinoma into four distinct molecular subtypes [7]. After the genomic, proteomic, and transcriptomic characterization of 373 cases, they classified endometrial carcinoma into four groups: *POLE* ultra-mutated, microsatellite instability hypermutated, copy-number low, and copy-number high, which offers a more objective and reproducible classification and has been shown to have prognostic and therapeutic implications [7]. Approximately 80% of endometrial cancers in the United States are endometrioid cancer, which is primarily hormonally driven and arises from a precursor lesion, endometrial hyperplasia.

## 2. Precursor to Endometrial Cancer

### Endometrial Hyperplasia

Endometrial hyperplasia (EH) is a condition characterized by a crowded, proliferative endometrium that may lead to or exist alongside endometrial carcinoma (EC). EH arises from estrogen stimulation and the presence of relatively less progesterone. Ferenczy et al. [8] distinguished EH from EC by using a histological lens and classified EH as the presence of glandular proliferation with no cytologic atypia, whereas endometrial intraepithelial neoplasia (EIN) was defined as the presence of lesions with cytologic atypia.

The 1985 classification system by Kurman et al. [9] used both cytological atypia and architectural features to identify precursor lesions (atypical endometrial hyperplasia). The study classified endometrial proliferation with no cytologic atypia as “hyperplasia” and those with cytologic atypia as “atypical hyperplasia”. The next subdivision was based on the degree of glandular complexity and crowding. Those lesions with no atypia present and minimum-to-moderate glandular crowding were identified as “simple hyperplasia”. No atypia and more glandular crowding were identified as “complex hyperplasia”. If the lesions had cytologic atypia without glandular crowding, this was classified as “simple atypical hyperplasia”. If there were both cytologic atypia and glandular crowding, this was classified as “complex atypical hyperplasia”. The study reported a statistically significant difference in the risk of progression from endometrial hyperplasia to carcinoma between those with hyperplasia (2% risk) and those with atypical hyperplasia (23% risk) [9]. However, concerns have been raised over the variable amounts of diagnostic reproducibility in the different classification systems used by clinicians.

The World Health Organization’s (WHO) 1994 four-class classification system has since been revised and was simplified into two categories of hyperplasia [10,11]: (i) hyperplasia without atypia and (ii) atypical hyperplasia or EIN.

The new WHO classification system continues to be a widely accepted diagnostic approach, as the new schema provides a clear distinction between those clinico-pathologic groups that have their own management algorithms. The 2015 guidelines of the American College of Obstetricians and Gynecologists (ACOG) shifted the preferred terminology from “atypical endometrial hyperplasia” to “EIN” [12]. The evolution of new diagnostic subtypes, such as EIN, has been helpful in diagnosing pre-malignant lesions and their likelihood of progressing to malignancy. However, grading the degree of cytologic/nuclear atypia at biopsy has been shown to be highly predictive of the findings seen at hysterectomy. A study by D’Angelo et al. [12] reported high reproducibility within and between the observers for the diagnosis of low-grade and high-grade atypical endometrial hyperplasia (AEH).

The risk of EH increases according to the conditions associated with the proliferation of estrogen and relatively lower amounts of protective progesterone. For pre-menopausal women, EH is typically seen in the background of intermittent or absent ovulation, whereas post-menopausal women who suffer from EH have increased estrogen in the form of obesity or hormone replacement therapy. In a patient with abnormal uterine bleeding or post-menopausal bleeding with a thickened endometrial stripe (>4 mm) (transvaginal ultrasound) and the aforementioned risk factors, EH should be considered, and cancer needs to be ruled out with a biopsy. A diagnosis of EH can be made with either an endometrial biopsy or a dilation and curettage (D&C). Hysteroscopy with directed D&C is the preferred method, if feasible, to visualize and target lesions within the endometrium and provide the best opportunity to exclude cancer (ACOG [12]). In a Gynecologic Oncology Group (GOG) study, 289 hysterectomies were performed for EH; upon pathology review, 123 had concurrent endometrial cancer (42.6%) [13]. Staining for phosphatase and tensin homolog gene (PTEN) has been associated with high mutation rates, with a concurrent decrease in protein expression, which suggests that an inactivating mutation in the gene is relevant to concurrent endometrial cancer [7,14].

The treatment of EH without atypia is called progesterone-based therapy. When the relevant studies were reviewed, there was a 96% regression rate when a levonorgestrel intrauterine device was used. In patients with atypia, the patients should be referred to a gynecologic oncologist because of the high rate of concurrent EC, and a hysterectomy is recommended [15]. Due to the prevalence of this diagnosis in pre-menopausal women who desire fertility, and when considering those who are not surgical candidates, hormonal management is an option. Progestin-based therapy is the treatment of choice. No clinical trials have been performed to suggest a superior progestin, and either high-dose oral progesterone (medroxyprogesterone or megestrol acetate) or a levonorgestrel intrauterine device can be used [16]. The optimal dose of oral progesterone has not been established Trimble et al., 2006 [13]. Oral progesterone may cause more side effects, such as weight gain, edema, thrombophlebitis, depression, and occasionally hypertension. The risk of venous thromboembolism may also slightly increase.

For those who proceed with hysterectomy, recent studies have confirmed that patients should have surgical staging with a hysterectomy, bilateral salpingo-oophorectomy (BSO), and sentinel lymph node (SLN) mapping and biopsies because of the high concurrence of EC [17]. A minimally invasive surgery (MIS) approach should be offered if feasible. Recent studies have confirmed that SLN assessment appears to be safe and should be offered in the case that occult malignancy is detected [18]. If a decision is made for uterine retention, a levonorgestrel intrauterine device or 10 to 20 mg of Provera daily for 14 days a month in pre-menopausal women and daily for post-menopausal patients should be employed; if there is no response, the dose can be increased to 40 mg per day. Megace can be administered daily at a dose ranging from 160–320 mg daily [19]. The endometrium should be sampled for regression every 3 to 6 months, and a study by Kim et al. [20] showed that D&C is superior to endometrial aspiration. If there is no regression after two biopsies, a hysterectomy should be recommended. Lifestyle should be addressed in these women, as, commonly, obesity contributes to anovulation, and unopposed estrogen contributes to this hyperplasia [21].

## 3. Endometrial Cancer

### 3.1. Diagnosis and Work-Up

The most common presentation of EC is vaginal bleeding. Abnormal bleeding in a pre-menopausal woman who is over the age of 35 years and has other risk factors, such as anovulation, obesity, or any post-menopausal bleeding, should be investigated. An in-office endometrial biopsy can be performed, or a D&C. Currently, the ACOG recommends a hysteroscopy with directed D&C, if feasible, to visualize and target lesions within the endometrium, and this provides the best opportunity to exclude cancer [12]. If a fractional D&C is not performed, an endocervical curettage should be carried out to evaluate for disease in the cervix. An ultrasound can be performed first for post-menopausal bleeding, and if the endometrial lining is less than 4 mm, a biopsy does not need to be performed unless it is persistent (ACOG [12]). If an office biopsy is performed and is negative, but bleeding persists, a D&C needs to be performed because there is a 10% false negative rate. A diagnosis of EC will be associated with a grade. There are three grades to assess the glandular component: grade 1 represents a solid non-squamous growth pattern of less than or equal to 5%; grade 2, a 6 to 50% solid non-squamous growth pattern; and grade 3, a greater than 50% solid non-squamous growth pattern [22]. Endometrial cancer spreads via a direct extension to adjacent structures, the transtubal passage of exfoliated cells, lymphatic dissemination, and hematogenous dissemination.

Once a diagnosis of EC is made, if high-risk pathology is present (serous, clear cell carcinosarcoma, grade 2–3 endometrioid), a computed tomographic (CT) scan of the chest, abdomen, and pelvis should be performed to rule out metastatic disease [23]. A cancer antigen (CA)-125 level can also be obtained in high-risk cases, as elevation can correlate with metastatic spread [24]. Early-stage disease is amenable to a surgical staging procedure, which is discussed in the treatment section later. The presence of advanced disease is an indication for neoadjuvant chemotherapy. Table 1 shows the current International Federation of Gynecology and Obstetrics (FIGO) staging for EC.

### 3.2. Prognostic Indices

The stage of disease is most significant for overall prognosis; however, age is an independent prognostic marker [25]. Histology, grade, myometrial invasion, lymphovascular space invasion (LVSI), tumor size, peritoneal cytology, hormone receptor status, ploidy and markers, body mass index (BMI), and the therapy received are other prognostic markers. The Surveillance, Epidemiology, and End Results (SEER) database from the National Cancer Institute (NCI) showed that women with EC who were less than 40 years old had improved survival when compared to those who were older than 40 years, regardless of stage [26].

In regard to histology, of all the endometrial cell types, serous carcinomas have a poor prognosis, even if there is no myometrial invasion or lymph node metastasis. They are often concurrently present in endometrioid tumors, and if they are present in more than 25% of the tumor, this tends to lead to a poor prognosis. Clear cell carcinomas are a rare type of EC (less than 5%) but commonly have LVSI and are also considered to have a high-grade histology [27]. When clear cell or serous carcinoma is completely removed with a curettage, the prognosis is not poor [28]. As the grade and myometrial invasion increase, there is more chance of lymph node metastasis and a worse prognosis. In a GOG study looking at stage I endometrial carcinomas, those that were grade 1 and only had inner, one-third myometrial invasion only had a 3% incidence of pelvic lymph node metastasis, but grade 3 lesions with outer, one-third myometrial invasion had an incidence of 34% [29].

The LVSI in EC is an independent risk for recurrence and death, regardless of histologic type, with a relative risk of death of 1.5 per incident found in a GOG study [25]. In stage I disease, there is a 15% incidence, which is even higher if there is deep myometrial invasion and a higher grade. There is also a risk for parametrial involvement and lymph node metastasis in early-stage EC when LVSI is identified [30]. In the PORTEC 1 and 2 trials, it was found that substantial LVSI, as opposed to focal, was the strongest risk factor for pelvic recurrence, distant metastasis, and overall survival [31].

Tumor size contributes to nodal disease, and if it is greater than 2 cm, it is associated with nodal involvement in high-risk endometrial cancer [32]. The Mayo Clinic incorporated a tumor size of greater or less than 2 cm into their scoring system in terms of the risk of lymph node metastasis in endometrioid endometrial cancer [33].

Peritoneal cytology was previously part of the FIGO staging, as it was thought to worsen prognosis and increase the risk of recurrence [34], but recent studies have shown that the poor prognosis associated with malignant pelvic washings can be attributed to other adverse prognostic indicators [35,36]. Those patients with positive estrogen and progesterone receptors (more so progesterone than estrogen) have an independent prognosis for longer survival [37].

Aneuploidy is the strongest marker for poor prognosis and can increase the risk of early recurrence and death, with an estimated relative risk of 4.1 for disease-related death [38]. In addition, the genetic markers that are associated with poor prognosis and death are the loss of beta-catenin expression and the loss of PTEN, and in patients with early-stage disease, this incurs a worse prognosis [39]. Recently, the TCGA proposed four distinct EC molecular subgroups based on mutational burden, microsatellite instability (MSI), and copy number alterations, as observed in 373 endometrial cancer cases: copy-number high, copy-number low, MSI hypermutated, and polymerase epsilon gene (*POLE*) ultra-mutated [Table 2] [40,41]. A higher BMI also plays a role, and an increased BMI is associated with an increase in all-cause mortality in endometrial cancer [42].

Finally, treatment does have a bearing on survival outcomes, and patients who received hysterectomy or hysterectomy-plus-radiation did better than those patients who received radiation alone [43].

## 4. Treatment

Similar to endometrial hyperplasia, the standard treatment for suspected early-stage endometrial cancer is surgical with a total hysterectomy, and BSO is recommended when the operation is feasible in those who are medically appropriate. The results from the Laparoscopic Approach to Cancer of the Endometrium (LACE) trial suggest that, unless this is not possible (large uterus, pelvic mass, significant adhesive disease, or cannot tolerate steep Trendelenburg), the procedure should be performed using an MIS approach to decrease potential morbidity, re-admission, and re-operation rates [44,45].

Lymph node evaluation should be carried out at the time of surgery to assign a complete surgical stage and for prognostic purposes to treat women with positive nodes and to direct adjuvant radiation therapy when needed. Unless an open approach is required, pelvic lymphadenectomy for surgical staging has fallen out of favor. Currently, for MIS procedures, if the equipment is available, the Society of Gynecologic Oncology (SGO) recommends SLN mapping with the cervical injection of indocyanine green (ICG) and biopsies, unless the lymph nodes do not map, in which case a pelvic lymphadenectomy should be performed [46]. This allows for a decrease in complications from complete lymphadenectomies while still obtaining prognostic information on node status [46]. Randomized controlled trials were used to evaluate SLN biopsies versus lymphadenectomies for all stage 1 endometrial cancers, and it was found that there is high diagnostic accuracy in detecting endometrial cancer metastases, and this can replace pelvic lymphadenectomy endometrial cancer staging [47,48]. The SLN biopsy will not identify metastases in 3% of patients with node-positive disease but protects patients from the morbidity of lymphadenectomy, which is acceptable [48].

A gross evaluation of the uterus or frozen section can be performed at the time of surgery and is reportedly 78 to 98.2% accurate [49,50]. The risk of lymph node metastases in patients with grade 1 or 2 endometrioid EC that are less than 2 cm and invade less than 50% of the myometrium is less than 1%, whereas those with deep myometrial invasion (intermediate risk) have a 9% risk of positive lymph nodes [51,52]. The SLN biopsies should be performed on patients with tumors greater than 2 cm, grade 3 lesions, clear cell or serous carcinoma, greater than 50% myometrial invasion, and cervical extension. If either the pelvic or para-aortic lymph nodes appear enlarged or bulky, then they should be removed at the time of surgery. Otherwise, para-aortic lymph nodes should not routinely be removed, and studies have shown that if the pelvic lymph nodes are negative, 96.2% of patients have negative para-aortic lymph nodes [53,54]. An omental biopsy should be performed in high-risk EC and when there appears to be suspicious lesions [55].

### 4.1. Stage I

#### 4.1.1. Endometrioid

Patients who are low risk with FIGO stage IA (grade 1 or 2) do not require adjuvant therapy and can be observed post-operatively as there is a low risk of recurrence, as determined by the initial PORTEC trial, which showed this to be the case in patients younger than 60 years [56]. Those patients who are 60 years of age or older and have LVSI are at higher risk and should receive vaginal brachytherapy [57,58].

Patients at high–intermediate risk, stage 1A (grade 3) or stage 1B (grades 1 or 2), should receive vaginal brachytherapy to reduce the risk of vaginal recurrence, as determined by the PORTEC 2 trial [59]. Patients with stage 1B, grade 3 should receive external beam radiation, which is derived from a meta-analysis from five randomized trials; this showed that there was a 10% survival advantage for patients with this high of a risk of lymph node involvement for stage 1 disease [60]. Systemic therapy in patients with high-risk early-stage disease is controversial but can be considered. PORTEC 3 (patients with stage 1b, grade 3 endometrioid, II or III endometrioid, or stage 1 to III serous or clear cell) evaluated concurrent radiotherapy plus cisplatin followed by carboplatin plus paclitaxel (versus radiotherapy alone), and from this, it was concluded that for these high-risk patients, the addition of chemotherapy did not improve overall survival (OS) but did increase failure-free survival (FFS) [61]. GOG-249 was a randomized trial that looked at adjuvant chemotherapy with carboplatin and paclitaxel and vaginal cuff brachytherapy versus pelvic radiotherapy in a lower-risk group of patients (than those enrolled in PORTEC 3). The authors found that there was no difference in relapse-free survival (RFS) or OS, suggesting that pelvic radiotherapy alone is adequate for stage I or II endometrial cancer, which is in line with the PORTEC 3 data [62]. Table 3 summarizes the treatment by stage for endometrioid EC.

#### 4.1.2. Serous and Clear Cell

Vaginal brachytherapy reduces the risk of vaginal recurrence in all non-endometrioid ECs, but most recurrences occur distantly [63]. Due to this risk of distant recurrence, even for stage 1 disease, treatment with adjuvant platinum and taxane-based therapy is commonly performed; however, there are no randomized control trials to support this. In PORTEC 3, for the patients with clear cell and serous histology who received chemotherapy, there is a similar FFS as there is in endometrioid histology [61]. A multicenter study showed that there was better local control and disease-free survival (DFS) in patients with stage 1A clear cell or serous EC who received adjuvant chemotherapy and radiotherapy [64]. A National Cancer Database (NCDB) study showed that chemo-radiation was associated with improved survival for all stages of serous EC, and this was confirmed by a recent meta-analysis showing that combined chemotherapy and radiotherapy significantly reduced the risk of death compared to chemotherapy alone [65,66]. A study by Einstein et al. [67] showed that a pilot study of 84 patients who received three cycles of carboplatin and paclitaxel followed by brachytherapy and three more cycles of chemo in sandwich therapy was well tolerated and was effective in patients with completely resected serous endometrial cancer. However, in contrast, there are prospective studies that show no difference to adjuvant chemotherapy in serous and clear cell histology. Ultimately, it is a risk versus benefit discussion with patients based on the above information and clinical experience.

### 4.2. Stage II

#### Endometrioid

There are no randomized prospective clinical trials for stage II disease, but the SEER data have been used to determine that treatment for clinical stage II disease should include a radical hysterectomy as it showed increased survival. Surgical staging should also be performed. If the patient is not suitable for primary surgery, external beam radiotherapy and brachytherapy can be performed, and if the tumor shrinks and the patient becomes a surgical candidate, surgical staging with a simple hysterectomy can be performed thereafter. Adjuvant therapy after surgical staging consists of external beam radiotherapy and vaginal brachytherapy or systemic therapy and can be considered as per the PORTEC 3 trial, which is outlined above and is similar to stage 1B, grade 3 cancers [61].

### 4.3. Stage III

Stage III disease can be discovered after surgical staging or can be noted pre-operatively through imaging. If it is diagnosed upon surgical staging, adjuvant treatment should be used with systemic chemotherapy and consideration of radiotherapy, as has been shown in PORTEC 3 [61]. Studies have shown that approximately 40% of patients with positive para-aortic nodes after surgical staging can have DFS with the addition of extended-field radiation therapy, and this is superior to chemotherapy alone, with positive nodes found at the time of surgery [68].

### 4.4. Stage IV

Usually, stage IV disease is noted through pre-operative imaging and is not usually surgically resectable. It is best treated with neoadjuvant chemotherapy, carboplatin, and taxane-based agents. If there is a response to chemotherapy, there are retrospective data that show that cytoreductive surgery can be beneficial [69]. A major point in the treatment of this stage of disease is to achieve local disease control in the pelvis in order to control bleeding and pain if present, whether this is carried out through palliative surgery to remove the uterus or palliative radiation to control bleeding.

There are limited studies that guide the treatment of advanced-stage EC, and further research is needed. As biological markers are discovered and tested for therapeutic benefits, there will continue to be more options available to such patients. In particular, serous uterine cancers can see over-expression in terms of human epidermal growth factor receptor 2 (HER2), and although this confers a poor prognosis, it is a targetable mutation with an anti-HER2 drug, and Trastuzumab can be added to standard chemotherapy in advanced disease, allowing for improved survival outcomes [70].

## 5. Special Considerations

### 5.1. Endometrial Cancer Diagnosed after Hysterectomy

If a patient is referred after a hysterectomy and, incidentally, is found to have a malignancy, whether a work-up was not performed or whether the pre-op biopsy missed the cancer, the patient will require re-operation to remove the adnexa (if not already removed) and a lymph node evaluation. If it is a high-risk histology, a CT evaluation should be performed before returning to the operating room to rule out distant or widespread metastasis [71].

### 5.2. Synchronous Primary Tumors

Data from the SEER database showed that women with synchronous ovarian and endometrial cancer were younger, had stage IA, grade I disease, and, if the ovary was also stage IA, survival was similar to that of stage 1A endometrial cancer. Often, the histology for endometrial and ovarian tumors are similar, and the assumption should be made that the tumors are separate primaries for treatment purposes unless metastasis can be proven [72].

### 5.3. Endometrial Cancer and Fertility Sparing in Young Women

Endometrial cancers in young women are generally well-differentiated and limited to the endometrium [73]. Many times, fertility is desired, and a good first step is to obtain pelvic magnetic resonance imaging (MRI) to assess for myometrial invasion. If there is no invasion and tumors are grade 1, it is safe to proceed with medical management using progestin therapy, which is similar to that outlined earlier for endometrial hyperplasia. The Royal College of Obstetricians and Gynecologists (RCOG [74]) did a study that showed that progestins can cause the regression of these early-stage tumors in 70 to 85% of cases. A small study showed that if oral megestrol or medroxyprogesterone were used and a complete response was achieved, recurrence saw a median of 3.5 years [75]. The levonorgestrel intrauterine device can also be considered, especially since it has fewer systemic side effects; however, some studies have shown that it may be less effective than oral progestins [76]. The ovaries should be imaged to ensure there is not a concurrent ovarian primary, but if normal, it can be considered for preservation and does not affect survival and actually improves survival due to a decreased risk of cardiovascular death [77]. There is no data for long-term management for these patients, and currently, the recommendation is for hysterectomy after childbearing is complete [78].

### 5.4. Lynch Syndrome

This syndrome is caused by mutations in DNA that mismatch repair genes (*MLH1, MSH2, MSH6*, and *PMS2*) and causes 2–5% of endometrial cancers, and patients are also at risk of colon cancer, urologic cancer, and breast cancer. In 50% of cases, endometrial cancer is the presenting cancer, and patients with this mutation have a 35 to 60% lifetime risk of developing endometrial cancer. Although there is no consensus screening for endometrial cancer, experts suggest that patients should be screened at age 30 to 35 years with transvaginal ultrasound and endometrial biopsy and should have an endometrial biopsy throughout their lives if any abnormal bleeding is reported. Patients should have risk-reducing surgery with a hysterectomy once their child-bearing stage is complete [79]. All patients who have a diagnosis of endometrial cancer under the age of 60 years should have routine immune-histochemistry to search for mismatch repair genes and single-gene sequencing when protein expression is not present [80].

### 5.5. Follow-Up

After treatment, visits should be scheduled every 3 months for the first year, every 4 months for the second year, and then every 6 months until 5 years is reached. After 5 years, they can be seen by a regular gynecologist but need to report any abnormal symptoms, especially vaginal bleeding, as 10% of recurrences occur after 5 years [81]. A pelvic exam should be performed at every visit, and if any abnormality is palpated, a biopsy should be taken if amenable, or a CT scan should be obtained if this is not feasible or if other concerning symptoms are reported.

### 5.6. Recurrent Endometrial Cancer

As of 2021, the 5-year relative survival rate for women diagnosed with EC is over 80% [82]. While most patients with EC who are diagnosed at an early stage are successfully treated using either surgery or adjuvant treatment, 7–15% of early-stage patients still experience recurrence [83]. Those patients with advanced disease at diagnosis have a 5-year survival rate of less than 50% for those with lymph node metastases and less than 20% for those with peritoneal or distant metastases [84]. The standard of care for advanced, metastatic, and recurrent EC has been systemic chemotherapy with paclitaxel plus carboplatin [85]. The addition of bevacizumab has been shown to increase the overall response rate from 54% to 72.7% and increase the PFS from 8.7 months to 13 months [86].

In terms of the site of recurrence, 4–20% of early-stage and up to 50% of late-stage EC have para-aortic or pelvic lymph node recurrence [87]. Pelvic and para-aortic nodal recurrence typically occurs in patients who have not undergone the appropriate surgery and/or external beam radiation. These patients may achieve long-term survival with chemotherapy alongside dose escalation using intensity-modulated radiation therapy [88]. Isolated vaginal recurrences have the most favorable response to radiation therapy. In a multi-institutional study in the United States, patients with surgical stage I EC with isolated vaginal recurrence and without prior adjuvant radiation were controlled and had an 81% control rate of vaginal recurrence under the use of radiation therapy [89]. The combination of external beam radiation therapy and high-dose brachytherapy has been shown to improve local control but is associated with the development of distant metastases, which leads to a 5-year OS of less than 50% [90]. If a patient has a vaginal recurrence and prior radiation therapy, an exenteration may offer the only possible cure [91]. If a vaginal recurrence is diagnosed, it is important to use positron emission tomography (PET)/CT imaging to exclude other sites of disease. Systemic recurrences are most common in high-grade histologies and those with advanced-stage disease who have a poor prognosis. Patients with stage IVB disease have a 20% 5-year survival [29]. Certain patients with completely resectable recurrent disease may benefit from a surgical approach using salvage cytoreductive surgery for recurrent EC, as it is associated with longer post-recurrence survival when compared to those with gross residual disease [92]. However, those who present with distant recurrent EC are typically treated from a palliative approach of systemic therapies, such as chemotherapy or hormonal therapy [83].

Hormonal therapy plays a large role in the treatment of advanced and recurrent EC, and objective response rates (ORR) of 15–20% have been reported. Patients with progesterone-positive tumors who have recurrent EC may benefit from hormonal therapy with medroxyprogesterone acetate. From a study that looked at high and low doses of this medication, the GOG recommended 200 mg/day [93]. Those who initially responded to progestins and present with recurrence may consider tamoxifen, a first-generation selective estrogen response modulator [93]. If there is a response to treatment, then treatment can continue indefinitely unless the response stops or side-effects occur. Tamoxifen, a selective estrogen response modulator and anti-estrogen medication, is used in recurrent EC and has a response rate of 22% [94]. It can also be continued for as long as the patient is responding to the treatment. Aromatase inhibitors can also be used, and their response rate is 10% [95]. The PARAGON trial, which looked at treatment with Anastrozole for women with estrogen and progesterone receptor-positive recurrent EC, showed a 44% clinical response but only 7% ORR [96]. With the addition of a mechanistic target of rapamycin (mTOR) inhibitor to an aromatase inhibitor using letrozole and everolimus, there was a response rate of 35% [97].

Chemotherapy is also a treatment option for patients with recurrent EC, but its intent is palliative. The most common and effective agents are platinum- and taxane-based, as well as anthracyclines. The response rates range from 30–60% for recurrent disease and a PFS of 5 to 14 months [98]. The combination of paclitaxel and carboplatin has resulted in response rates as high as 67% [99]. The MITO trial added bevacizumab to this regimen and increased the OS rate from 54% to 72.7%, with an improvement in PFS from 8.7 to 13 months [86]. In those patients who have recurrence after receiving carboplatin and paclitaxel, there is a paucity of data with which to recommend therapy, but some data suggests that it should be treated similarly to ovarian cancer, and if enough time has occurred in between the therapies, an additional trial of this therapy should be attempted [100]. The response rate to second-line chemotherapy for recurrent EC is poor, and the best response rate is with taxane, with the GOG reporting a 35% response rate in previously untreated patients [101]. Other chemotherapies have been studied as second-line agents, such as doxorubicin and gemcitabine, with low-to-no response rates [98].

The available treatment options for recurrent EC were once limited, but newly emerging targeted therapies based on tumor characteristics and molecular profiles are starting to gain momentum. Programmed death 1 (PD-1) is an immune checkpoint receptor that interacts with programmed death ligand 1 (PD-L1) on tumor-infiltrating T-cells. PD-1 has become a focus for immunotherapy by inactivating its interaction with PD-L1, thus preventing T-cell-mediated tumor cytolysis [84,102,103]. In the context of endometrial carcinoma, it has been demonstrated that 83% of primary endometrial tumors and 100% of metastatic endometrial tumors express PD-L1 [104]. Pembrolizumab is a PD-1-blocking antibody with beneficial anti-tumor activity that has been demonstrated to be efficacious in the treatment of PD-L1-positive solid tumors [103].

Pembrolizumab immunotherapy is currently approved for a subset of microsatellite instability-high (MSI-H) or mismatch repair deficient (dMMR) endometrial cancers, which make up only 13–30% of recurrent EC cases [105,106]. On the other hand, almost 84% of cases of recurrent ECs have microsatellite-stable (MSS) or microsatellite-indeterminate tumors. A treatment regimen involving pembrolizumab and lenvatinib (a multi-kinase inhibitor) is a promising approach for those with MSS, disease progression after prior systemic therapy, or recurrent EC [107]. The high-risk subtypes of EC, such as clear cell, serous, and grade 3 endometrioid histology, represent only 28% of cases yet around 74% of deaths because of the extensive rate of recurrence [108]. As mentioned previously, the dysregulation or amplification of Her2/neu occurs in 15–30% of serous and 16% of clear cell EC and is another potential approach to targeted therapy [109,110]. Fader and colleagues [109] conducted a phase II trial and compared the PFS time among patients with stage III or IV uterine serous carcinoma when given carboplatin-paclitaxel (control) versus carboplatin and paclitaxel with trastuzumab. The patients in the control group who received first-line therapy had a PFS of 9.3 months, whereas those in the experimental group had a PFS of 17.9 months [109].

## 6. Conclusions

Endometrial cancer is the most common gynecologic malignancy, with rising incidence and mortality. Significant racial disparities and geographic subpopulations also exist for this disease. Most recently, endometrial cancer has been subclassified into four different categories that dictate prognosis and therapeutic interventions. Currently, it is surgically staged unless advanced disease and stage determine the prognosis as well as the treatment. The cornerstone of early-stage disease is radiotherapy to prevent vaginal cuff recurrence, whereas advanced-stage and recurrent disease are typically treated with chemotherapy and targeted hormonal therapy. Most recently, targeted immune-onco-therapy has been studied for advanced and recurrent disease, seeing improved survival. Further research and clinical trials to explore additional treatment options, including immune and targeted therapies for patients with advanced and recurrent endometrial cancer, are ongoing, with hopes of prolonging survival and finding cures for patients with this disease.

## Figures and Tables

**Table 1:** 2009 FIGO surgical staging for endometrial carcinoma.

Stage I ^a^	Tumor confined to the corpus uteri
IA ^a^	No or less than half myometrial invasion
IB ^a^	Invasion equal to or more than half of the myometrium
Stage II ^a^	Tumor invades cervical stroma but does not extend beyond the uterus ^b^
Stage III ^a^	Local and/or regional spread
IIIA ^a^	Tumor invades the serosa of the corpus uteri and/or adnexa ^c^
IIIB ^a^	Vaginal and/or parametrial involvement ^c^
IIIC ^a^	Metastases to pelvic and/or para-aortic lymph nodes ^c^
IIIC1 ^a^	Positive pelvic lymph nodes
IIIC2 ^a^	Positive para-aortic lymph nodes with or without positive pelvic lymph nodes
Stage IV ^a^	Tumor invades bladder and/or bowel mucos and/or distant metastases
IVA ^a^	Tumor invasion of bladder and/or bowel mucosa
IVB ^a^	Distant metastases, including intra-abdominal metastases and/or inguinal lymph nodes

^a^ Either Grade 1, Grade 2, or Grade 3. ^b^ Endocervical glandular involvement should only be considered as stage I and no longer as stage II. ^c^ Positive cytology has to be reported separately without changing the stage.

**Table 2:** Characteristics of the TCGA molecular classification of endometrial cancer [40,41].

Type	*POLE* (Ultra-Mutated)	Microsatellite Instability (MSI) (Hypermutated)	Copy Number Low (Endometrioid)	Copy Number High (Serous Like)
Prevalence	7%	28%	39%	26%
Mutation frequency	Very high (>100 mutations/Mb)	High 100–10 mutations/Mb	Low <10 mutations/Mb	Low <10 mutations/Mb
Commonly mutated genes	*POLE* (100%), *PTEN* (94%)	*PTEN* (88%) *PIK3CA* (54%)	*PTEN* (77%) *CTNNB* (52%)	*TP53* (92%) *PIK3CA* (47%)
Copy number aberrations	Very low	Low	Low	High
MSI/*MLH1* methylation	Mixed high and low MSI, stable	High MSI (*MLH1*, *PMS2*, *MSH2*, and/or *MSH6* deficiency)	MSI stable	MSI stable
Histological subtype	Endometrioid	Mostly endometrioid	Endometrioid	Serous, 25% high-grade endometrioid and mixed
Grade	G1–3	G1–3	G1–2	G3
Other features	Ambiguous histo-morphology Dense immune infiltrates	Display tumor-infiltrating lymphocytes	*CTNNB* mutations are associated with poor prognosis. Subgroup with amplification of chromosome arm 1q has poor prognosis	Similar to high-grade serous ovarian carcinoma L1 cell adhesion molecule (L1CAM) expression associated with poor prognosis
Prognosis	Good	moderate	moderate	Poor

**Table 3:** Treatment by stage for endometrioid endometrial cancer.

FIGO 2009 Stage	Grade	Surgery	Adjuvant Therapy
IA	1, 2	TAH/TLH + BSO + SLN	Observation preferred, consider vaginal brachytherapy if LVSI or age ≥ 60.
			Vaginal brachytherapy preferred.
	3	TAH/TLH + BSO + SLN	Consider observation if no myo-invasion.
			Consider EBRT if age ≥ 70 or LVSI.
IB	1	TAH/TLH + BSO + SLN	Vaginal brachytherapy preferred.
			Consider observation if age <60 and no LVSI.
			Vaginal brachytherapy preferred.
	2	TAH/TLH + BSO + SLN	Consider EBRT if age ≥ 60 or LVSI.
			Consider observation if age <60 and no LVSI.
	3	TAH/TLH + BSO + SLN	EBRT and/or vaginal brachytherapy ± systemic therapy (category 2b).
II *	1, 2, 3	TAH/TLH or radical hysterectomy + BSO + SLN	EBRT (preferred) and/or vaginal brachytherapy ± systemic therapy (category 2b)
III *	1, 2, 3	TAH/TLH or radical hysterectomy +BSO + SLN	Systemic therapy
			± EBRT
			± brachytherapy
IV */**	1, 2, 3		Systemic therapy
			± EBRT
			± brachytherapy

* For locoregional disease for which surgery is not feasible, give EBRT and/or systemic therapy and re-evaluate for surgery. ** For distant metastasis, re-evaluate for surgical resection and/or radiation based on response. Abbreviations: TAH = total abdominal hysterectomy; TLH = total laparoscopic hysterectomy; BSO = bilateral salpingo-oophorectomy; SLN = sentinel lymph nodes; EBRT = external beam radiation; LVSI = lympho-vascular space invasion.
